# Molecular Subtyping of Cancer Based on Distinguishing Co-Expression Modules and Machine Learning

**DOI:** 10.3389/fgene.2022.866005

**Published:** 2022-05-02

**Authors:** Peishuo Sun, Ying Wu, Chaoyi Yin, Hongyang Jiang, Ying Xu, Huiyan Sun

**Affiliations:** ^1^ School of Artificial Intelligence, Jilin University, Changchun, China; ^2^ Phase I Clinical Trails Center, The First Affiliated Hospital, China Medical University, Shenyang, China; ^3^ Computational Systems Biology Lab, Department of Biochemistry and Molecular Biology and Institute of Bioinformatics University of Georgia, Athens, GA, United States; ^4^ Key Laboratory of Symbol Computation and Knowledge Engineering of Ministry of Education, Jilin University, Changchun, China

**Keywords:** molecular subtyping of cancer, specific co-expression module, network perturbation, multi-classification, machine learning

## Abstract

Molecular subtyping of cancer is recognized as a critical and challenging step towards individualized therapy. Most existing computational methods solve this problem *via* multi-classification of gene-expressions of cancer samples. Although these methods, especially deep learning, perform well in data classification, they usually require large amounts of data for model training and have limitations in interpretability. Besides, as cancer is a complex systemic disease, the phenotypic difference between cancer samples can hardly be fully understood by only analyzing single molecules, and differential expression-based molecular subtyping methods are reportedly not conserved. To address the above issues, we present here a new framework for molecular subtyping of cancer through identifying a robust specific co-expression module for each subtype of cancer, generating network features for each sample by perturbing correlation levels of specific edges, and then training a deep neural network for multi-class classification. When applied to breast cancer (BRCA) and stomach adenocarcinoma (STAD) molecular subtyping, it has superior classification performance over existing methods. In addition to improving classification performance, we consider the specific co-expressed modules selected for subtyping to be biologically meaningful, which potentially offers new insight for diagnostic biomarker design, mechanistic studies of cancer, and individualized treatment plan selection.

## 1 Introduction

Precision cancer medicine aims to characterize the distinct biology of an individual or a group of cancer patients sharing certain commonalities and treat them by targeting the specific oncogenic event shared by such a group (Lipinski et al., 2016; Russnes et al., 2017; Ozturk et al., 2018; Zhang et al., 2019). Using breast cancer as an example, the majority of such cancers fall into one of the three subtypes: estrogen receptor positive (ER+), human epidermal growth factor receptor 2 positive (HER2+), and triple-negative (Vuong et al., 2014). Distinct treatment plans have been developed to effectively treat these three subtypes of breast cancer. Patients with ER+ tumors receive endocrine therapy, supplemented with chemotherapy for some; patients of HER2+ tumors receive targeted drug therapy or small-molecule inhibitor therapy combined with chemotherapy; and patients of triple-negative breast cancer are treated using chemotherapy only (Waks and Winer, 2019; Yin et al., 2020). Clearly, the effectiveness of such a treatment plan depends on our ability to accurately subtype cancer tissues with shared biology, particularly common druggable targets among subgroups of a specific cancer type (Chaisaingmongkol et al., 2017). This is the focus of the current study, specifically to identify distinguishing features, measured using transcriptomic data, only shared by samples of each specified subtype of cancer (Valle et al., 2020).

Cancer subtyping through applications of machine learning techniques has been done by numerous authors on multiple cancer types. Cascianelli et al. developed a classification method for breast cancer subtyping that employs several machine learning classifiers to solve the multi-classification task for breast cancer subtyping (Cascianelli et al., 2020). Markus et al. modeled and solved the breast cancer subtyping problem based on integrated analyses of gene expression and DNA methylation data using a random forest algorithm (List et al., 2014). Deep-learning algorithms have recently been applied to tackle the cancer subtyping problem through an end-to-end approach. Guo, et al. have reported a deep-learning framework to learn the representation of high-dimensional features derived from gene expression data and alternative splicing profiles and solve the subtyping problem of breast cancer (Yang et al., 2018).

While these methods, such as deep learning, have powerful capabilities in data classification, most of these methods have limitations in interpretability and tend to require large amounts of data for model training (Chen et al., 2019), which has clearly limited the applications of omic-data based subtyping. In addition, these methods generally rely on gene expression data for classification and have largely ignored the interaction information among the expressed genes in cancer, which generally carries more information than the expression levels of individual genes (Segura-Lepe et al., 2019; Lee et al., 2020). This is particularly important for modeling genes in cancer tissues, knowing that considerable metabolic reprogramming has taken place in cancer tissue cells, as we have previously demonstrated (Sun et al., 2020), which could be captured by co-expression information. Hence, it is worth the effort to develop co-expression-based classifiers to capture the distinct reprogrammed metabolisms and hence the corresponding phenotypes of individual subtypes of cancer.

A few papers have been published on cancer subtyping based on co-expression information, which classify cancer samples based on the general characteristics of the relevant co-expression networks (Liu et al., 2016; Yu et al., 2020). Jiang et al. developed a multi-classification method for cancer samples based on differential co-expression analyses (Jiang et al., 2019), and predicted a sample’s label through calculating its perturbation on the most specific edges of each subclass-representing network module. Although this method performs well in cancer subtyping, there is a lack of interpretability as the identified edges tend to be unconnected, hence the lack of functional information.

In this paper, we present a new cancer molecular subtype classification framework based on a specific co-expression module and a deep neural network (DNN) named SCM-DNN, which can identify a robust, distinct co-expression module for each subtype of a cancer. A co-expression module is a set of genes whose expressions highly correlate with each other (Wolf et al., 2014), and a distinguishing co-expression module is a co-expression module that is associated with a specific subtype but not other subtypes of a cancer. Intuitively, a distinguishing co-expression module should reflect certain unique characteristics of a cancer subtype. Specifically, we use the TCGA transcriptomics data to construct a co-expression network over samples of each subtype and then apply weighted correlation network analysis (WGCNA) (Zhang and Horvath, 2005; Langfelder and Horvath, 2008; Sipko et al., 2018) to partition the network into co-expression modules. Then we assess the discerning power of each co-expression module for cancer subtyping by (1) identifying the most discerning modules and their most specific edges between samples of the current subtype and samples of other subtypes; 2) perturbing the correlation levels of such edges to generate new samples with co-expression network features for each sample; and 3) then training the classifier based on such new samples. When applying this classifier to breast cancer (BRCA) and stomach adenocarcinoma (STAD), we found it has superior performance under both macro-average recall (Macro-R) and macro-average f1-score (Macro-F1) metrics over existing methods. We consider that this co-expression module-based subtyping not only provides an improved method for cancer subtyping but also provides meaningful information about the unique biology of cancer samples of each subtype, hence potentially offering new information about the underlying mechanism of the cancer subtype and suggesting new individualized treatment targets.

## 2 Materials and Methods

**FIGURE 1:**
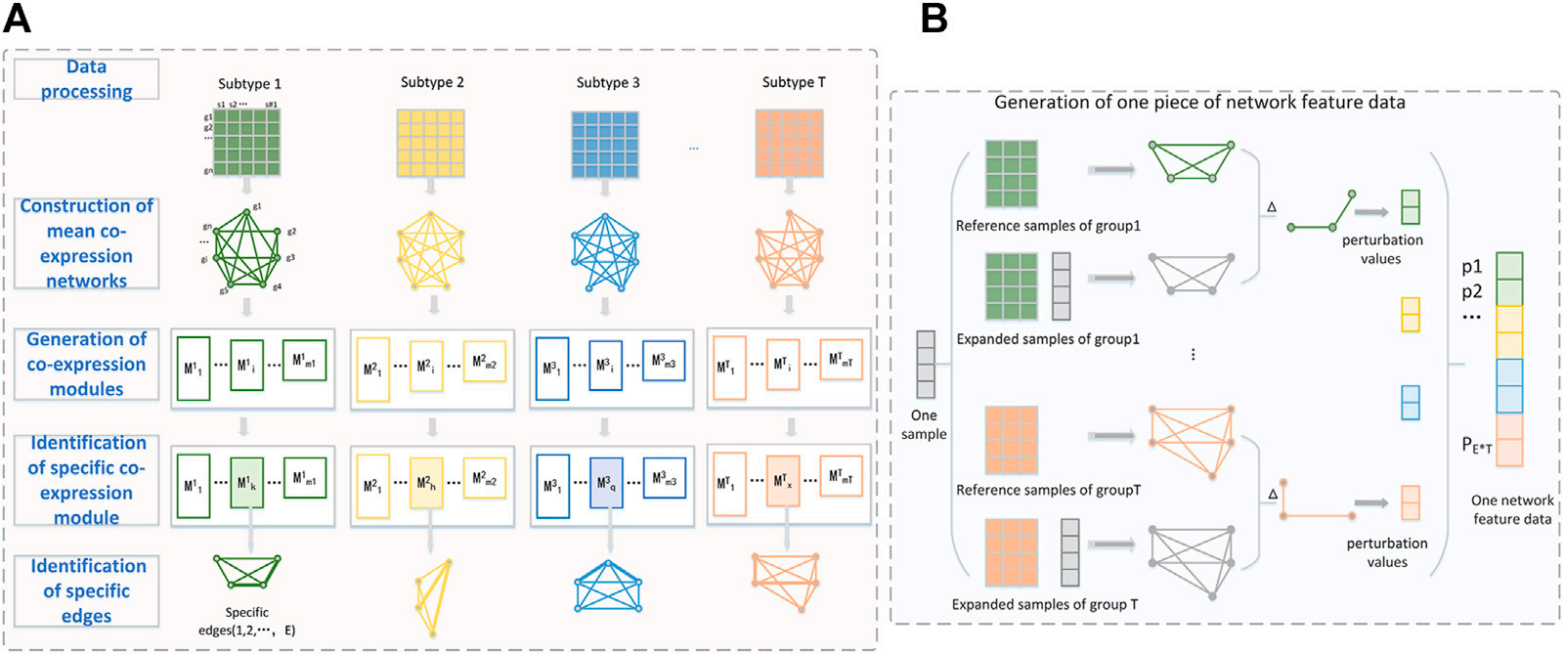
**(A)** The workflow from data processing to specific edges identification. Take four-subclass classification as an example. Each subtype is represented as a gene expression matrix with n genes after data processing. WGCNA is used to divide whole gene set into different co-expression modules. The specific edges of one subtype are extracted from the specific module of their subtype. The perturbation of these specific edges (gene pairs) is used to generate network features data. **(B)** Detailed process of generating one piece of network feature data. The perturbation values of a sample are the difference of specific edges between expanded network and the reference network.

**FIGURE 2:**
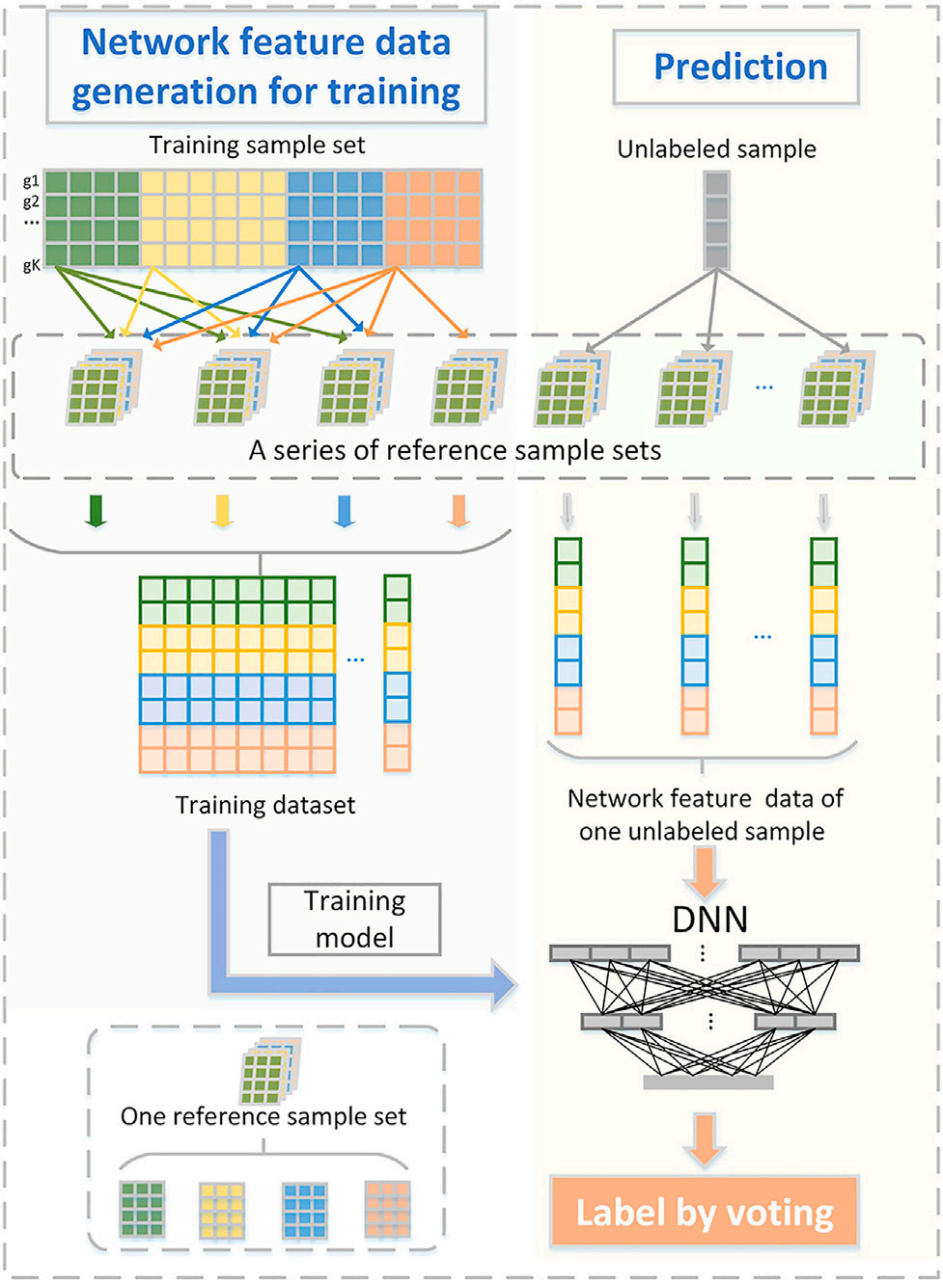
Sufficient network feature data generation for model training and prediction. One reference sample set consists of T groups of samples that from T subtype (T: total number of subclass). Network feature data corresponding to training samples are used for model training.

We present a new computational framework, SCM-DNN, shown in Figure 1 and Figure 2, for subtyping cancer samples.

### 2.1 Data Processing

RNA-seq data and clinical information of breast cancer and stomach cancer tissue and normal samples are downloaded from the TCGA database (Weinstein et al., 2013). These cancer samples are pre-labeled with their subtype information. Overall, 113, 437, 37 and 115 samples are labeled as control, ER+, HER2+, and triple-negative BRCA tissues respectively; and 33, 107, 23, 47, and 50 samples are marked as control, CIN, EBV, MSI, and GS STAD tissues, respectively. The FPKM value (with log2 transformation) is used to measure the expression levels in our analysis. For each cancer type, genes whose average expression levels are less than 10 over all the samples are removed, and the median absolute deviation (mad) is used to estimate the variance of a gene’s expression. In a dataset with sample size N, the ‘mad’ value of gene X is calculated as follows:

$$mad=median\left(\left|X_{i}-median\left(X\right)\right|\right)\left(i=1,2,\ldots ,N\right).$$ (1)

*X* = (*X*_1_, *X*_2_, … , *X*_*i*_, … , *X*_*N*_), *X*_*i*_ is the expression value of gene X of the *i*th sample. Clearly, the more similar the expression levels of a gene are across all samples, the closer its “mad” value is to zero. For our analyses, we only keep the top 90% genes with the largest “mad” values. Overall, 14,439 and 7,761 genes are kept for BRCA and STAD, respectively.

### 2.2 Construction of Co-Expression Networks and Generation of the Co-Expression Modules

For each cancer type, we first construct gene co-expression networks for each subtype; that is, for a cancer type with T molecular subtypes, T co-expression networks need to be constructed. The Spearman correlation coefficient is used to construct the co-expression networks. According to (Anglani et al., 2014), although spearman correlation is an efficient way to construct co-expression networks, its coefficient and statistical significance depend on the sample size to some extent. Since the issue of imbalanced sample size always exists, directly constructing co-expression networks for each category will lead to incomparability among different categories. To solve this problem, we perform sampling to construct the co-expression network for each cancer type.

Given the sample sizes of each subtype {*s*1, *s*2, ...*sT*}, we have performed F-fold sampling to calculate the correlations for each subset, with each fold having *N*_*s*_ samples. *N*_*s*_ should be smaller than min {*s*1, *s*2, ...*sT*}, and F should be large enough to ensure that all samples are selected at least one time. For the *f*th fold in *l*th subset, $cor_{f}^{l}$ represents the correlation values matrix for the co-expression network, and $p_{f}^{l}$ represents the corresponding *p*-values. The final correlation values and *p*-values of *l*th subset are defined as Formula (2) and (3):

$$cor^{l}=\frac{1}{F}\overset{F}{\underset{f=1}{\sum }}cor_{f}^{l}.$$ (2)

$$P^{l}={\left(\overset{F}{\underset{f=1}{\prod }}p_{f}^{l}\right)}^{1/F}.$$ (3)

Furthermore, we have removed gene pairs in the network whose associations are not significant (i.e., *p*-value >0.01) and genes that do not connect with any other genes in the network. In the end, we have obtained T co-expression networks {*MeanNet*1, *MeanNet*2, ...*MeanNetT*} for each subtype. For each MeanNet, we apply WGCNA to divide it into several co-expression modules. We set the soft thresholds according to the scale free topology fitting index R2 coefficient for each subtype. It reweights the MeanNet by adjusting the coefficient of each co-expression pair to make the network satisfy the scale-free property. All the genes are then hierarchically clustered into different groups based on the weighted network, and the genes that can’t cluster together with other genes are stored in Module0.

### 2.3 Identification of the Specific Co-Expression Modules

A specific co-expression module is defined if the genes of a subtype are highly correlated in a subtype but weakly correlated within other subtypes. It is worth noting that we don’t consider Module0 of each subtype. We identify the specific co-expression module of each subtype by integrating the following two scores:

Score 1: Specific aggregation score. If genes of one subset are concentrated in a module of one subtype but they are scattered in many different modules for all the other subtypes, it indicates that these genes have a specific co-expression pattern in this subtype. According to this idea, we perform a cross calculation among all the modules of different subtypes to evaluate the specificity of each module. For module $M_{i}^{s}\left(i=1,2,...,Sn\right)$ , we first get the gene intersections of $M_{i}^{s}$ and $M_{j}^{t}$ . (s: source subtype, $M_{i}^{s}$ : the *i*th module of subtype s, Sn: number of modules in the source subtype, $M_{j}^{t}$ : the *j*th module of subtype t, t: target subtype, *t* ∈ {1, 2, ..*T*}\*s*, T is the total number of subtypes). In order to avoid the bias caused by the number of genes in each module, we will calculate the overlap ratio between $M_{i}^{s}$ and $M_{j}^{t}$ as:

$$Overlapratio_{\left(s,t,i,j\right)}=\frac{\left|M_{i}^{s}\cap M_{j}^{t}\right|}{\left|M_{i}^{s}\right|}.$$ (4)

If for any t and j, the *Overlapratio*_(*s*,*t*,*i*,*j*)_ values of $M_{i}^{s}$ are small, it indicates that the genes in scarcely cluster together in other subtypes. So, for a module $M_{i}^{s}$ , we define $Max\;overlapratio_{i}^{s}$ to represent the maximal overlap between $M_{i}^{s}$ and all the other modules of other subtypes. Then, we sort all modules’ *Max overlapratio* of this subtype in ascending order and the ranking of $M_{i}^{s}$ is equal to its score 1. The lower ranking of $Max\;overlapratio_{i}^{s}$ , the more likely $M_{i}^{s}$ will be identified as a specific co-expression module.

Score 2: Correlation significance score. If co-expression coefficients of the edges in this module are overall significantly stronger than their coefficients in other module subtypes, then this module is more likely to be a specific one.

For a certain module $M_{i}^{s}$ , the mean co-expression value of its edges is defined as $edgemean_{M_{i}^{s}}^{s}$ . Meanwhile, the mean co-expression value of these edges on other subtypes’ co-expression networks is calculated and denoted as $edgemean_{M_{i}^{s}}^{t}$ (t: target subtype, *t* ∈ {1, 2, ..*T*}\*s*). If some edges in $M_{i}^{s}$ do not appear in co-expression network of subtype t, their values in subtype t are recorded as 0. Then the difference between $edgemean_{M_{i}^{s}}^{s}$ and is $edgemean_{M_{i}^{s}}^{t}$ is defined as:

$$△edgemean_{M_{i}^{s}}^{s,t}=edgemean_{M_{i}^{s}}^{s}-edgemean_{M_{i}^{s}}^{t}.$$ (5)

$△\min\;mean_{M_{i}^{s}}$ represents the smallest $△edgemean_{M_{i}^{s}}^{s,t}$ of $M_{i}^{s}$ . Next we sorted $△\min\;mean_{M_{i}^{s}}\left(i=1,2,\ldots S_{n}\right)$ in a descending order, their ranking is defined as score 2. Similarly, the lower rank $△\min\;mean_{M_{i}^{s}}$ is, the more likely $M_{i}^{s}$ is to be a specific co-expression module. Taking the sum of score 1 and score 2 as final score for each module $M_{i}^{s}\left(i=1,2,\ldots S_{n}\right)$ , we rearrange all modules of subtypes in an ascending order, and select the module with lowest rank as the specific co-expression module of subtype s.

### 2.4 Identification of Specific Edges in Specific Modules

As the sizes of specific modules are different and there are many edges in each specific module, it is necessary for us to select the most specific edges that are highly co-expressed only in one subtype to represent the character of each specific module. In addition, selecting same number of edges for each subclass can improve the comparability. If we want to select E specific edges for each specific module, following steps can be taken. For a gene pairs (*i*, *j*) in the specific co-expression module, their correlation values on all subtypes are denoted as $\left(cor_{\left(i,j\right)}^{1},cor_{\left(i,j\right)}^{2},\ldots ,cor_{\left(i,j\right)}^{T}\right)$ (T is the number of subtypes), and $\max cor_{\left(i,j\right)}^{x}$ is the max value of $cor_{\left(i,j\right)}^{x}\left(x=1,2,\ldots ,T\right)\backslash s$ . Then, the difference between $cor_{\left(i,j\right)}^{s}$ and $\max\;cor_{\left(i,j\right)}^{x}$ is defined as:

$$△cor_{\left(i,j\right)}^{s}=cor_{\left(i,j\right)}^{s}-\max cor_{\left(i,j\right)}^{x}.$$ (6)

The $△cor_{\left(i,j\right)}^{s}$ of all gene pairs are sorted in descending order, and the top E gene pairs are specific edges.

### 2.5 Generation of Network Feature for Each Cancer Sample

Although specific co-expression modules could capture the prominent characteristics of each subtype, it is not easy to transfer these characteristics directly to a single sample. Hence, our method proposes learning the sample’s network feature by calculating its perturbation effect when adding it to each specific module. Intuitively, when a sample is added to the specific co-expression module of its same subtype, its disturbance to this module is not significant. Otherwise, when adding this sample to specific modules of other subtypes, their disturbance is relatively large.

For each subtype, we randomly select 90% of the samples as the training set and the remaining 10% as the test set. In order to avoid the classification bias due to imbalanced sample sizes of different subtypes, we generate and amplify new samples by adding one sample to multiple reference network sets and ensuring the sample sizes of each subtype are similar for training.

First, we generate a series of reference network sets covering the specific co-expression edges of each subtype. Reference network of one subtype is generated by genes in its specific module, naturally, specific co-expression edges are covered. The size of samples used for constructing reference networks is uniformly assigned as P (P is smaller than the sample size of any subtype). For each subsampling, a reference network set is generated, including T reference networks corresponding to T subtypes, and we randomly select P samples from each subtype several times and generate several reference network sets, shown in Figure 2.

Then, one cancer sample is added to a reference set, which is T reference networks, to construct T new co-expression networks, called expanded networks. The perturbation value of a specific edge is obtained by calculating the difference between an expanded network and a reference network.

$$△cor_{i}^{x}=|cor_{i}^{x^{\prime }}-cor_{i}^{x}|.$$ (7)

Here, i is the *i*th specific edge of subtype x, $cor_{i}^{x^{\prime }}$ and $cor_{i}^{x}$ are the correlation value of *i*th specific edge of subtype x in the expanded network and reference network, respectively. $△cor_{i}^{x}$ when a sample is added to the reference network, is perturbation value to *i*th specific edge. Then, for one cancer sample, it’s T *E perturbation values are merged into a vector, where E is the number of specific edges selected for each subtype, generating a piece of network feature data.

One piece of network feature data shows the characteristics of a sample at the co-expression network level. In order to augment the sample size, we add each training sample to several reference network sets. Hence, we can obtain enough network feature data for model training even though there are few cancer samples, which guarantees the classifiers are able to learn sufficient information for each subtype. For each test sample, it is also randomly added to the reference network sets to generate its corresponding new sample(s). It is worth noting that all the reference networks are constructed from samples of training sets.

### 2.6 Construction of Cancer Subtype Multi-Classifier

We build a fully connected feed forward neural network classifier with cross-entropy loss function.

$$L=-\frac{1}{N}\underset{i}{\sum }\overset{T}{\underset{c=1}{\sum }}y_{ic}\log\left(p_{ic}\right).$$ (8)

Here, the value of *y* depends on the true label of data *i*. Let *h* be a neural network, in which the activation function of hidden layers and output layer are ReLu and softmax, respectively. *p*_*ic*_ is the probability of the data i belonging to subtype c. N is the size of the data. The optimization algorithm is stochastic gradient descent. We apply an early stop strategy to avoid over-fitting in the training process and take 10-fold cross validation to verify the performance of the classification method. In prediction, when adding each testing sample into different reference networks, it generates several new samples and then gets multiple prediction labels, voting strategy are used to obtain final prediction label of this sample.

### 2.7 Baseline Methods

We compared our method, SCM-DNN with three traditional filter feature selection methods (Chi-square test, Analysis of Variance, and Mutual Information), and one state-of-the-art wrapper feature selection method, (HSIC-Lasso) following with DNN. In addition, we also compared our method with one of the few co-expression-based cancer subtyping methods. Moreover, we compared our method with one of the few co-expression-based cancer subtyping methods (SCP), which predicted a sample’s label through calculating its perturbation on the most specific edges of each subclass-representing network. In addition, we also compared our method with DeepCC, which is a deep learning-based framework integrating functional spectra quantifying activities of biological pathways for molecular subtyping of cancer (Gao et al., 2019).

## 3 Results

### 3.1 Statistic of Distinguishing Co-Expression Modules of Each Cancer Subtype

14439 and 7761 genes were used for the construction of co-expression networks for BRCA and STAD, respectively. We decompose the co-expression network into several modules for each cancer subtype. The number of co-expression modules for each cancer subtype, and the number of genes and edges in each specific co-expression module are shown in Table 1.

**TABLE 1:** Statistics of co-expression modules of each cancer subtype.

	Subtypes	#Edges of	#Modules	#Genes in	#Edges in
Cancer		Co-expression		Specific	Specific
Types		Network		Co-expression	Co-expression
				Module	Module
BRCA	ER+	18002953	37	123	7161
BRCA	HER2+	26774509	20	1834	810674
BRCA	Triple	17261163	32	1334	322232
	Negative				
BRCA	Control	54354208	65	698	241789
STAD	CIN	5155648	43	124	4483
STAD	EBV	12952861	52	75	2328
STAD	GS	11937803	29	789	262884
STAD	MSI	8714653	18	190	9780
STAD	Control	17645626	50	105	5330

### 3.2 Evaluation of the Discerning Power of the Co-Expression Module for Each Subtype

To evaluate the discerning power and stability of each co-expression module between each subtype and the samples of the other subtypes of a cancer, we have used accuracy, macro-average recall and macro-average F1-score to avoid possible issues created by imbalanced sample sizes among the subtypes, defined as follow.

$$Accuracy=\frac{\sum_{i=1}^{T}TP_{i}}{\sum_{i=1}^{T}{\#}_{i}}.$$ (9)

$$Macro-P=\frac{1}{T}\overset{T}{\underset{i=1}{\sum }}\frac{TP_{i}}{TP_{i}+FP_{i}}.$$ (10)

$$Macro-R=\frac{1}{T}\overset{T}{\underset{i=1}{\sum }}\frac{TP_{i}}{TP_{i}+FN_{i}}.$$ (11)

$$Macro-F1=\frac{2*Macro-p*Macro-R}{Macro-p+Macro-R}.$$ (12)

Here, *#*i is the sample size of *i*th group; TP is for true positives, FP for false positives, FN for false negatives, and TN for true negatives.

For BRCA subtyping, we have conducted two experiments by selecting the top 100 and top 200 distinguishing co-expressed edges from each co-expression module to evaluate their discerning power. Considering the relatively small sample size and the number of features, a neural network with two-hidden layers is employed to train a classifier, which has 50 and 10 nodes on the first and the second layer, respectively. We have compared the performance of our approach with six other published classifiers (see Methods), each employing the same number of features as our approach.

**FIGURE 3:**
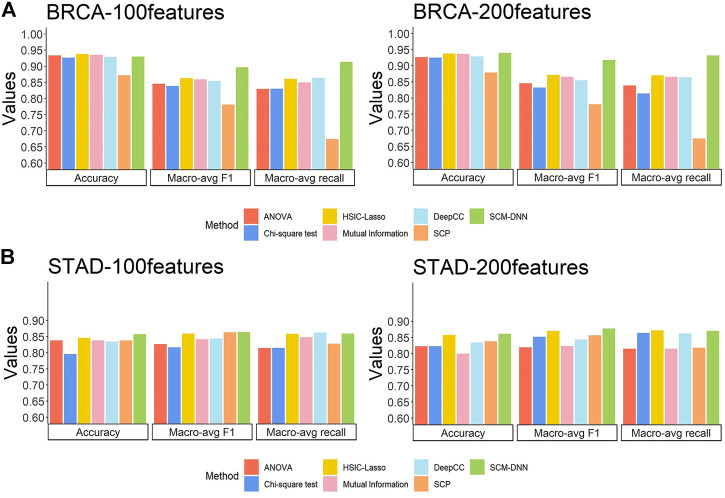
Cancer subtyping performance by seven methods: our method SCM-DNN,HSIC-LASSO, ANOVA, Chi-square mutual information, SCP and DeepCC **(A)** BRCA subtyping and **(B)** STAD subtyping with using top100 and 200 distinguishing co-expressed gene pairs.

The subtyping performance of our method on BRCA samples along with the performance by other five methods are shown in Figure 3A. Our method clearly performs better across all the metrics, especially in terms of macro-avg recall and macro-avg f1-score. Imbalanced sample sizes tend to create problems for classification methods, which tend to give higher weights to subtypes with higher numbers of samples. In BRCA, the numbers of samples for the four subtypes are 113, 437, 37, and 115, with HER2+ having the smallest sample size. We note that the recall values for HER2+ samples are 0.891, 0.675, 0.575, 0.475, 0.650, and 0.622 by SCM-DNN, HSIC-lasso, ANOVA, Chi-square, mutual information and DeepCC, respectively.

For STAD subtyping, we set the same experimental parameters, including the organization of the neural networks as for BRCA breast cancer molecular subtyping task. The performance by our method *vs*. the other six methods is comparable to that on BRCA, with our method performing the best as detailed in Figure 3B. It is worth noting that the DeepCC classified cancer samples according to a large number of genes which are not suitable for feature selection, so we use all its features and compared it with our method when selecting 100 features and 200 features, respectively.

Overall, the results reveal that our method gives the best and stable subtyping performance, particularly for the subtyping problems with highly imbalanced sample sizes. We found that our method always performs best specially in recall and F1-score, the reason is: we generate sufficient network feature data for neural network model training, and it avoids the situation that the classifier only learns sufficient information for the category with largest scale, instead of categories with small scale. Hence, our method is superior to other methods when predict the subtype with smallest scale. In addition, network feature data can reflect the characteristics of each individual subtypes. It also proves that specific modules with differentiation and robustness are conducive to improving classification performance. We display network feature data in the form of heat map and find that the samples of the same subtype naturally gather into one block. Details are shown in the Supplementary Material.

### 3.3 Functional Analyses of the Genes in Each Specific Module

To elucidate the possibly unique biology for each cancer subtype, a pathway enrichment analysis is conducted over edges of the identified co-expression module for each subtype. It is worth noting that the number of genes in specific modules of each molecular subtype is different. Specifically, there are 171, 86, 281 and 205 genes in the specific modules of control, ER+, HER2+ and triple negative BRCA samples, respectively, with detailed gene lists given in Supplementary Table S1. And their co-expressed gene pairs are selected for function analyses. The most significantly enriched biological processes and pathways enriched by each of the four gene sets are shown in Table 2.

**TABLE 2:** The most significantly enriched pathways by the genes belonging to top 200 specific edges of each molecular subtype in BRCA.

Pathway	*p*-Value
Controls	
GO:cell-cell adhesion	1.59E-05
KEGG:Regulation of actin cytoskeleton	9.65E-05
GO:leukotriene biosynthetic process	5.71E-04
GO:ephrin receptor signaling pathway	7.36E-04
KEGG:Cyanoamino acid metabolism	1.63E-03
KEGG:T cell receptor signaling pathway	1.97E-03
ER+	
GO:Wnt signaling pathway	5.94E-03
GO:negative regulation of Wnt signaling pathway	1.73E-02
GO:lens fiber cell development	2.68E-02
GO:positive regulation of DNA-templated	2.68E-02
transcription, initiation	
GO:epithelial cell-cell adhesion	3.43E-02
KEGG:HTLV-I infection	4.56E-02
GO:muscle organ development	4.66E-02
GO:eyelid development in camera-type eye	4.92E-02
HER2+	
GO:nitrobenzene metabolic process	1.15E-03
GO:substrate adhesion-dependent cell spreading	1.90E-03
GO:negative regulation of extrinsicapoptotic signaling pathway	1.90E-03
GO:skeletal system development	3.18E-03
GO:glutathione derivative biosynthetic process	3.42E-03
GO:outflow tract septum morphogenesis	3.90E-03
GO:xenobiotic catabolic process	3.91E-03
GO:positive regulation of cell migration	4.44E-03
Triple-negative	
GO:signal transduction	8.57E-07
GO:neuron migration	1.06E-04
GO:nervous system development	1.94E-04
GO:positive regulation of signal transduction	3.60E-03
KEGG:Thyroid hormone signaling pathway	4.16E-03
GO:positive regulation of phosphatidylinositol	4.52E-03
3-kinase signaling	
GO:cellular amino acid metabolic process	8.03E-03

The most enriched pathways in each distinct set of samples shown in Table 2 are quite informative. For example, pathways enriched by the control samples revealed key features of control *vs*. BRCA cancer samples in terms of their functionalities, namely cell-cell adhesion (which is altered in all cancer samples), interactions with immune cells (which is clearly altered in all cancer samples *vs.* controls). Similar can be said about neural functions (ephrin receptor signaling), cell polarity (which is considerably altered in cancer, actin cytoskeleton) and inflammation signaling (leukotriene biosynthesis). Similarly, the most enriched pathways for ER+ samples are growth related (Wnt signaling), muscle development (also including eyelid development and fiber cell development), and a specific type of immune response (HTLV-I infection). And the most enriched pathways for HER2+ are related to xenobiotic metabolism (including dealing with nitrobenzene), oxidative stress (glutathione biosynthesis), and cell morphogenesis changes. The pathways uniquely enriched by triple negative samples involve neural systems, a general indicator for the level of malignancy of a cancer type, and phosphatidylinositol 3-kinase signaling (a key regulator of cell polarity), also strongly indicating the level of malignancy of the cancer subtype.

For STAD, 72, 81,67,119, and 217 genes and their co-expressed gene pairs are selected as distinguishing features for the control, CIN, EBV, MSI, and GS STAD samples, respectively. The enrichment results by each gene set are shown in Table 3.

**TABLE 3:** The most significantly enriched pathways by the genes belonging to top 200 specific edges of each molecular subtype in STAD.

Pathway	*p*-Value
Controls	
GO:protein phosphorylation	8.83E-05
KEGG:Oxytocin signaling pathway	2.67E-04
GO:apoptotic cell clearance	1.64E-03
GO:peptidyl-serine phosphorylation	1.65E-03
KEGG:Endocrine and other factor-regulated	2.51E-03
calcium reabsorption	
GO:vesicle-mediated transport	3.35E-03
CIN	
GO:apoptotic process	8.53E-03
GO:steroid metabolic process	1.35E-02
GO:intracellular protein transport	1.61E-02
GO:catecholamine metabolic process	3.64E-02
GO:sulfation	4.43E-02
GO:response to toxic substance	4.78E-02
EBV	
KEGG:Metabolic pathways	5.84E-03
GO:response to ionizing radiation	1.16E-02
KEGG:Valine, leucine and isoleucine degradation	1.54E-02
GO:methylation	2.47E-02
GO:activation of cysteine-type endopeptidase activity	3.13E-02
involved in apoptotic process	
GO:mRNA splicing, *via* spliceosome	3.79E-02
MSI	
GO:immune response	3.05E-04
GO:response to interferon-gamma	4.37E-04
GO:type I interferon signaling pathway	6.88E-04
GO:interferon-gamma-mediated signaling pathway	1.02E-03
GO:inflammatory response	2.60E-03
GS	
GO:cell division	3.48E-08
KEGG:Cell cycle	9.42E-08
GO:mitotic nuclear division	2.59E-07
GO:mitotic nuclear envelope disassembly	7.55E-07
GO:sister chromatid cohesion	3.55E-06
GO:G2/M transition of mitotic cell cycle	5.18E-06

The distinct biology of each of the four subtypes of STAD samples, as indicated by their enriched pathways, is striking. For CIN subtype, we see strong indication of toxicity and detoxification in their cells, e.g., by response to toxic substance, sulfation, intracellular protein transport and steroid metabolic process. In EBV samples, the distinct characteristics are dealing with oxidative stress as shown by response to ionizing radiation, valine, leucine, and isoleucine degradation, activation of cysteine-type endopeptidase activity, and upregulation of spliceosome. In MSI, we see that all signals are related to inflammation and immune response in immune response, response to interferon-gamma, type I interferon signaling pathway, and inflammatory response. In GS, the key distinguishing characteristic is rapid cell division, as indicated by cell division, cell cycle, nuclear division, chromatid cohesion and G2/M transition.

### 3.4 Comparison of Selected Features Between Gene Expression Based and Co-Expression Based Methods

**FIGURE 4:**
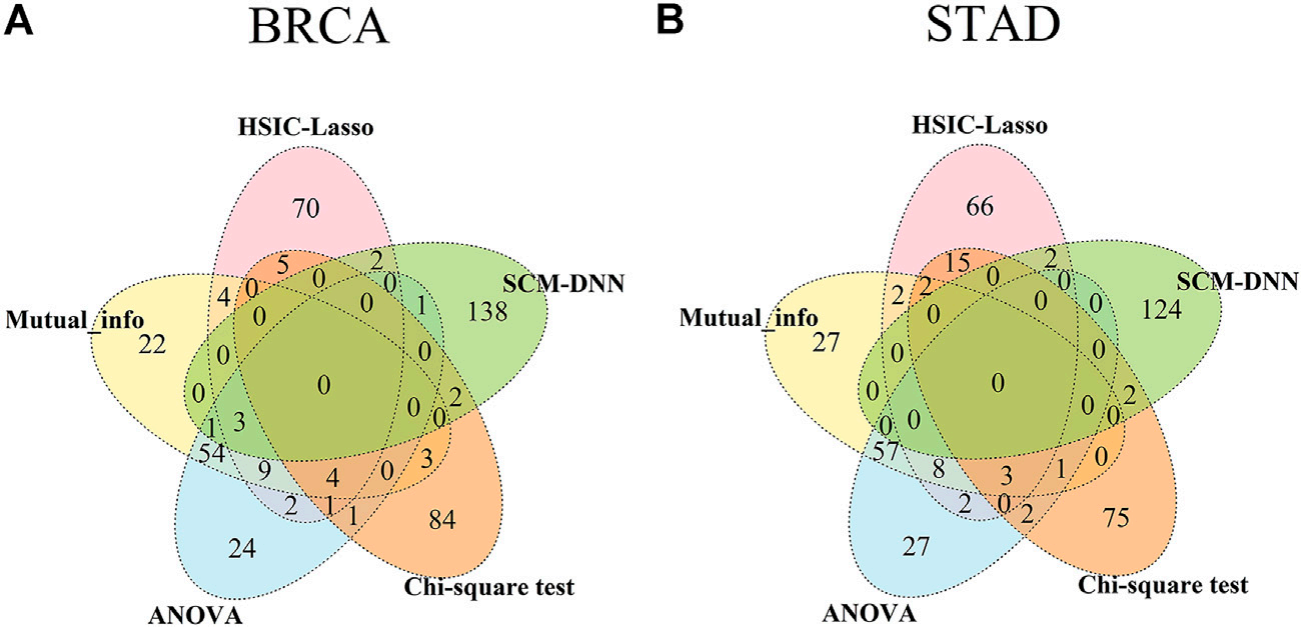
Venn diagram for overlaps among top 100 (network) features obtained by SCM-DNN, HSIC-LASSO, ANOVA, Chi-square and mutual information in **(A)** BRCA and **(B)** STAD.

We have compared the consistency and differences among the top 100 selected features obtained by each of the five methods, including ours, with results summarized in Figure 4. We note that genes selected based on gene-expression levels are quite different from the genes identified based on co-expression levels for both BRCA and STAD. And there is considerable overlap among the features selected by different gene-expression level-based methods. For example, genes selected by ANOVA and the mutual information method have a 60% overlap in both cancer types. It should be noted that the top 100 network features obtained by SCM-DNN are 100 gene-pairs, hence the number of genes for SCM-DNN is larger than 100.

Through further performing differential gene expression analyses on the genes obtained by SCM-DNN, we find their expression have little changes among different subtypes of the same cancer type. This result reveals that differential gene expression-based methods have clear limitations in characterizing changes in biological systems. Hence co-expression-based analyses for cancer subtyping and possibly many other cancer omic data analysis problems could prove to be the way to go.

We have also analyzed the connectivity of the selected genes in the co-expression modules. In our subtyping prediction, we used only the top 100 and 200 co-expressed gene pairs. An interesting observation is that all the selected genes could be connected using at most two additional genes in the relevant module, suggesting that the selected feature genes are strongly functionally associated. However, regarding the genes selected by traditional gene expression based feature selection methods, they are generally highly dispersed across a co-expression module.

Additionally, due to the transmissibility of information in a network, it’s not hard to control the whole module by managing a few nodes. Moreover, since these modules are specific to each molecular subtype, in other words, they are probably the most striking features of this disease. Hence, they are expected to be the most effective drug targets for individualized therapy.

## 4 Discussion

In this paper, we proposed a computational classification method for cancer molecular subtyping based on co-expression network features of each cancer sample. It has been recognized that the phenotypic difference in cancer samples can hardly be fully understood by only analyzing single molecules, and it is the relevant system or specific network that is ultimately responsible for such a phenomenon (Liu et al., 2016). Moreover, network-based biomarkers, e.g. subnetwork markers (Ideker and Krogan, 2012), network biomarkers (Liu et al., 2014), and edge biomarkers (Zhang et al., 2015), are demonstrated superior to traditional single molecule biomarkers for accurately characterizing disease states. However, it is generally challenging to construct specific network and obtain individual network feature for each sample (Liu et al., 2016). Here, we generate a sample’s network feature by calculating its perturbation effect on each background class-specific module after adding it to them. Intuitively, the quality of constructed class-specific networks will direct influence the generation of network feature and then further guide the final classification results. Hence, to ensure the robustness of each subtype specific network, we construct multiple co-expression networks for each molecular subtype by sampling and then integrate them. Our previous study had proved that sampling-based co-expression network construction could avoid the bias caused by both data noise and imbalanced sample size among different subtypes (Jiang et al., 2020). Class-specific modules are identified by a top-down approach (i.e. decomposing the whole co-expression network of each subtype and making comprehensive comparison across different subtypes), which is different from some existing specific modules identification method based on collecting specific co-expression gene pairs. In comparison, co-expression modules give a relatively complete path of signal transmission or transcriptional regulation, and provide much more information for us to understand biological mechanism of each subtype, and then could help researchers to identify both actionable targets for drug design as well as biomarkers for response prediction.

The classification performance of our method is superior to conventional molecule biomarker-based methods, when applied to breast and stomach cancer molecular subtyping, under several evaluation indexes. It is a universal framework and is expected to perform well in molecular subtyping task for other cancer types. Besides, it is also easy to transfer to other subtyping tasks, such as cancer sample staging and grading classification. Similarly, through constructing co-expression networks and extracting specific co-expression modules for each cancer stage or grade, a sample could be accurately classified according to its network features generated by calculating the perturbation effect of this sample on each background class-specific module. We assume that specific module of each cancer stage (or grade) can capture the essential distinguishing property of its samples. And adding a sample of a different class to the specific module will induce large disturbance, while adding a sample of its same class will not disturb too much. One of the advantages of this study is that it doesn’t need too many training samples. Prior knowledge in the basis of satisfying the statistical significance indicates that the sample number of each subtype reaching 15 is enough to construct co-expression networks for each subtype. Then, a large number of new samples with a network feature can be generated.

Omics data have enabled the unbiased characterization of the molecular features of multiple human diseases, particularly in cancer. Multi-omics may provide molecular insights beyond the sum of individual omics, and it is becoming increasingly common to characterize multiple omics layers to gain biological insights spanning multiple types of cellular processes (Vitrinel et al., 2019). Hence, in our further work, besides transcriptomics data, we will introduce other omics data to construct heterogeneous correlated networks and extract heterogeneous specific modules for each subtype. Moreover, this study provides a general framework with extensible and replaceable executive function modules. Other machine learning methods could be applied for the final multi-class classification according to specific task and data distribution.

## 5 Conclusion

We present here a new framework, SCM-DNN, to identify each molecular subtype’s robust, specific co-expression modules that could efficiently and steadily predict patients’ molecular subtypes of breast and stomach cancer. Compared with traditional gene expression based feature selection methods for multi-classification, SCM-DNN performs better under all the metrics even the sample size of each class is extremely imbalanced. Additionally, these specific genes identified by SCM-DNN could probably represent the striking characteristics of individual subtypes; meanwhile, they are concentrated in the co-expression network. Hence, they are promised to assist us to better understand the underlying mechanism of molecular subtyping and potentially guide individualized medicine.

Multi-omics data and their integration are recognized as an effective way to explore the biological mechanism. In future studies, we will make full use of those data to develop a more comprehensive and robust classification method by integrating multi-omics data to construct subtype-specific correlation networks for molecular subtyping of cancers, expecting a deeper mechanism to be discovered.

## Data Availability

The original contributions presented in the study are included in the article/Supplementary Material, further inquiries can be directed to the corresponding authors.
