# Supplementary material for: Molecular Subtyping of Cancer Based on Distinguishing Co-Expression Modules and Machine Learning
Source: Front Genet. 2022 May 2;13:866005. doi: 10.3389/fgene.2022.866005 (PMC9108363; doi:10.3389/fgene.2022.866005)
Supplement: Supplementary file 1 [file DataSheet1.PDF]

Supplementary Material

1 SUPPLEMENTARY FIGURES

Our algorithm is based on a key point:adding a sample of a different class to the specific module will induce large disturbance, while adding a sample of its same class will not disturb too much.In our study, we calculated the perturbation values of samples on the specific edges, and generated network feature data for model training.We found that these network feature data indeed conform to the above-mentioned law.Then,We used heat maps to characterize these data.See the figureS1 and figureS2 for details.

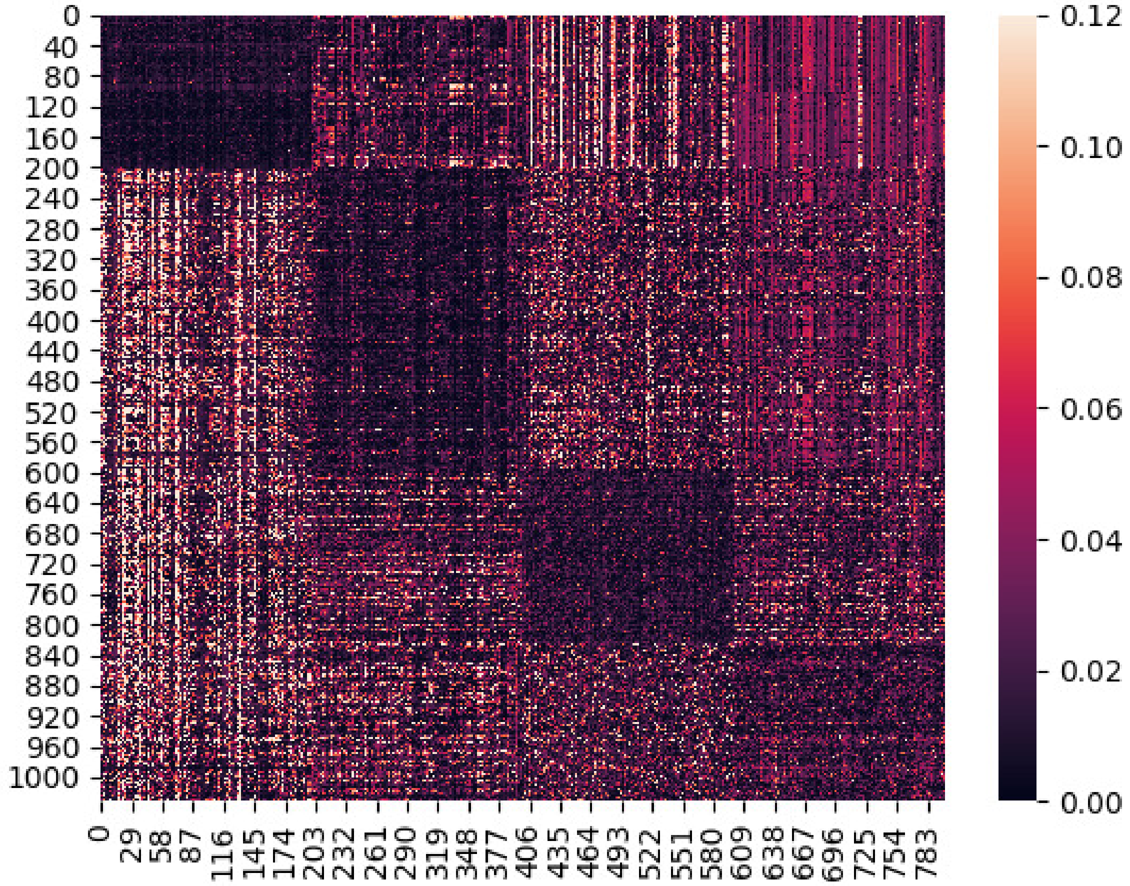

Figure S1. Heatmap of training network feature data for BRCA.

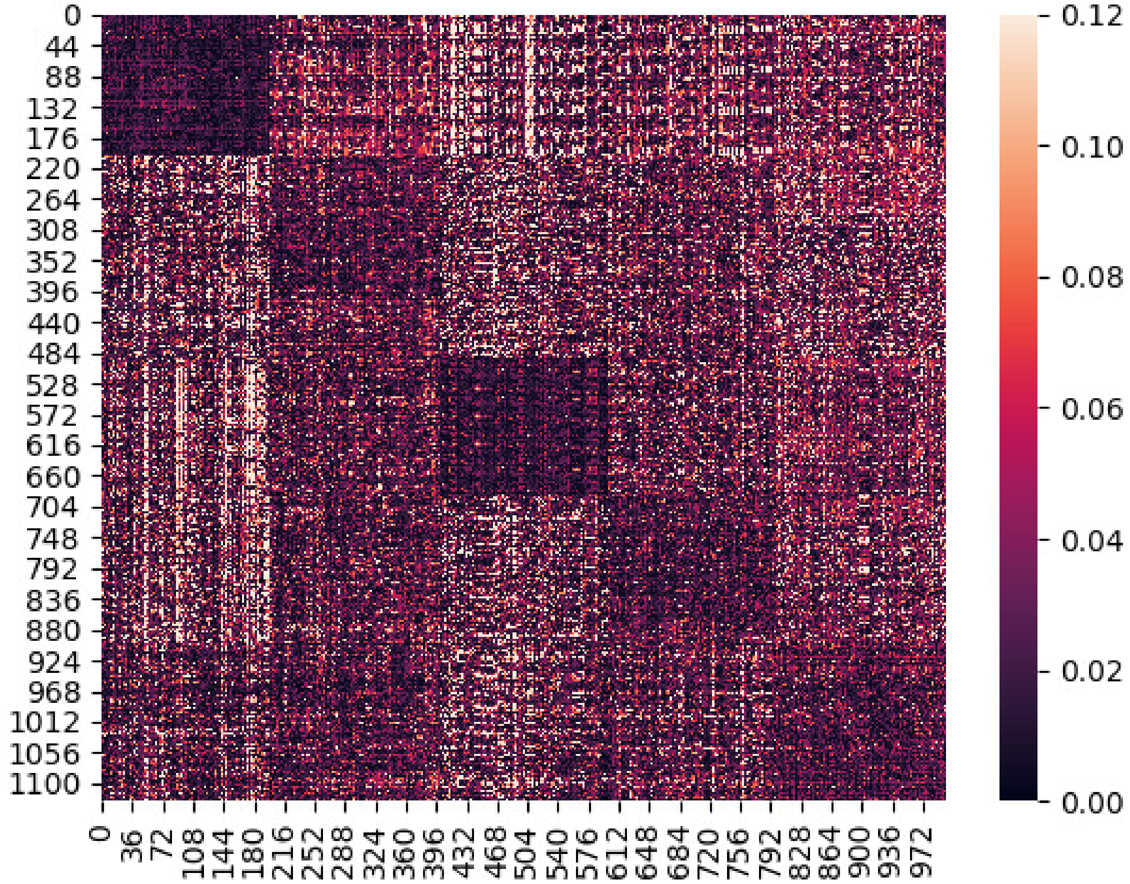

Figure S2. Heatmap of training network feature data for STAD.
